# High tension in sarcomeres hinders myocardial relaxation: A computational study

**DOI:** 10.1371/journal.pone.0204642

**Published:** 2018-10-04

**Authors:** Lauren J. Dupuis, Joost Lumens, Theo Arts, Tammo Delhaas

**Affiliations:** 1 Department of Biomedical Engineering, Cardiovascular Research Institute Maastricht (CARIM), Maastricht University, Maastricht, The Netherlands; 2 Université de Bordeaux, LIRYC L'Institut de Rythmologie et Modélisation Cardiaque, Bordeaux, France; York University, CANADA

## Abstract

Experiments have shown that the relaxation phase of cardiac sarcomeres during an isometric twitch is prolonged in muscles that reached a higher peak tension. However, the mechanism is not completely understood. We hypothesize that the binding of calcium to troponin is enhanced by the tension in the thin filament, thus contributing to the prolongation of contraction upon higher peak tension generation. To test this hypothesis, we developed a computational model of sarcomere mechanics that incorporates tension-dependence of calcium binding. The model was used to simulate isometric twitch experiments with time dependency in the form of a two-state cross-bridge cycle model and a transient intracellular calcium concentration. In the simulations, peak isometric twitch tension appeared to increase linearly by 51.1 KPa with sarcomere length from 1.9 μm to 2.2 μm. Experiments showed an increase of 47.3 KPa over the same range of sarcomere lengths. The duration of the twitch also increased with both sarcomere length and peak intracellular calcium concentration, likely to be induced by the inherently coupled increase of the peak tension in the thin filament. In the model simulations, the time to 50% relaxation (*t*_*R50*_) increased over the range of sarcomere lengths from 1.9 μm to 2.2 μm by 0.11s, comparable to the increased duration of 0.12s shown in experiments. Model simulated *t*_*R50*_ increased by 0.12s over the range of peak intracellular calcium concentrations from 0.87 μM to 1.45 μM. Our simulation results suggest that the prolongation of contraction at higher tension is a result of the tighter binding of *Ca*^*2+*^ to troponin in areas under higher tension, thus delaying the deactivation of the troponin.

## Introduction

Cardiac muscle contraction is triggered by the rise in the concentration of intracellular calcium [1]. It has been shown in skinned muscle preparations that a relatively small increase in the free intracellular calcium concentration, i.e. [*Ca*^*2+*^], results in disproportionately large increases in steady state tension development, a process that has been called cooperativity in sarcomere activation and contraction [2, 3]. In literature, researchers generally use a Hill coefficient (*n*_*Hill*_) to quantify cooperativity by determining the steepness of the slope in the [*Ca*^*2+*^]-tension relationship. Sun et al. have found that cooperativity is on the order of *n*_*Hill*_ = 3 under zero load conditions [4]. The data of Dobesh et al. has shown that in the presence of mechanical load, *n*_*Hill*_ approaches a value of 7 [2]. This increase of cooperativity suggests that tension in the thin filaments promotes the cooperative effect during sarcomere contraction.

Recently with the MechChem model, we introduced the hypothesis that tension in the thin filament tightens the binding of *Ca*^*2+*^ to the troponin complex (*Tn*) [5]. Model simulations showed that high tension boosts the cooperativity from the purely chemical cooperativity (*Ca*^*2+*^ binding to *Tn* in the absence of a mechanical load) shown by Sun et al. [4] to the cooperativity shown by Dobesh et al. [2] when the thin filament is under tension. The agreement between simulations and experimental data [5] suggested that the proposed tension-dependence of cooperativity may be responsible for the added cooperative activation of *Tn*’s.

Experiments have been conducted to release the afterload during an isometric twitch resulting in a quick drop in sarcomere tension [6, 7]. The drop in sarcomere tension corresponds with an instantaneous increase in the free intracellular calcium concentration. The immediate increase in free intracellular calcium concentration with a sudden decrease in sarcomere tension shows that the mechanics of the sarcomere has a clear influence on the binding of *Ca*^*2+*^ to *Tn*. Hence, it is likely that the tension-dependent activation of the *Tn*’s along the thin filament has an effect on the relaxation of cardiac muscle. It has been shown by Janssen and Hunter [8] that the duration of muscle contraction increases linearly with the developed peak tension. While Janssen and Hunter proposed that higher peak tension caused the longer duration of contraction, the underlying physical mechanism that explains the experimental results was lacking. In the present study, we hypothesize that prolongation of contraction, or delay in relaxation, is a result of hindrance to thin filament deactivation imposed by high tension locally in the thin filament. In order to test this hypothesis, we extend the MechChem model, originally designed to simulate steady state tension, with a two state cross-bridge cycle model and a transient intracellular calcium concentration. The additions to the model allow us to simulate isometric twitch contractions. We simulate an isometric twitch at multiple sarcomere lengths (*L*_*sarc*_) and compare the model results to the experimental data presented by Janssen and Hunter [8]. Additionally, we simulate an isometric twitch with varying peak intracellular calcium concentrations to mimic the experimental conditions of Kassiri et al [9].

## Methods

The previous version of the MechChem model was developed to describe a steady state isometric contraction. In that model, we assumed that *Ca*^*2+*^ binding to the *Tn* results in the movement of tropomyosin (*Tm*) out of the position in which it blocks the binding sites on the thin filament. The binding of *Ca*^*2+*^ to *Tn* is cooperative in nature with a baseline chemical cooperativity that is increased by mechanical tension in the thin filament. Additionally, it has been assumed that unblocking of cross-bridge (*XB*) binding sites implies automatic *XB* formation and force generation. Furthermore, we assumed that all *XB*’s form only in the single overlap region and generate equal force. Tension in the thin filament increases at each location of *XB* binding by the corresponding *XB* force. Thus, tension increases step by step from the beginning of the single overlap region near the center of the sarcomere towards the ending of this region near the z-disk.

**Fig 1:**
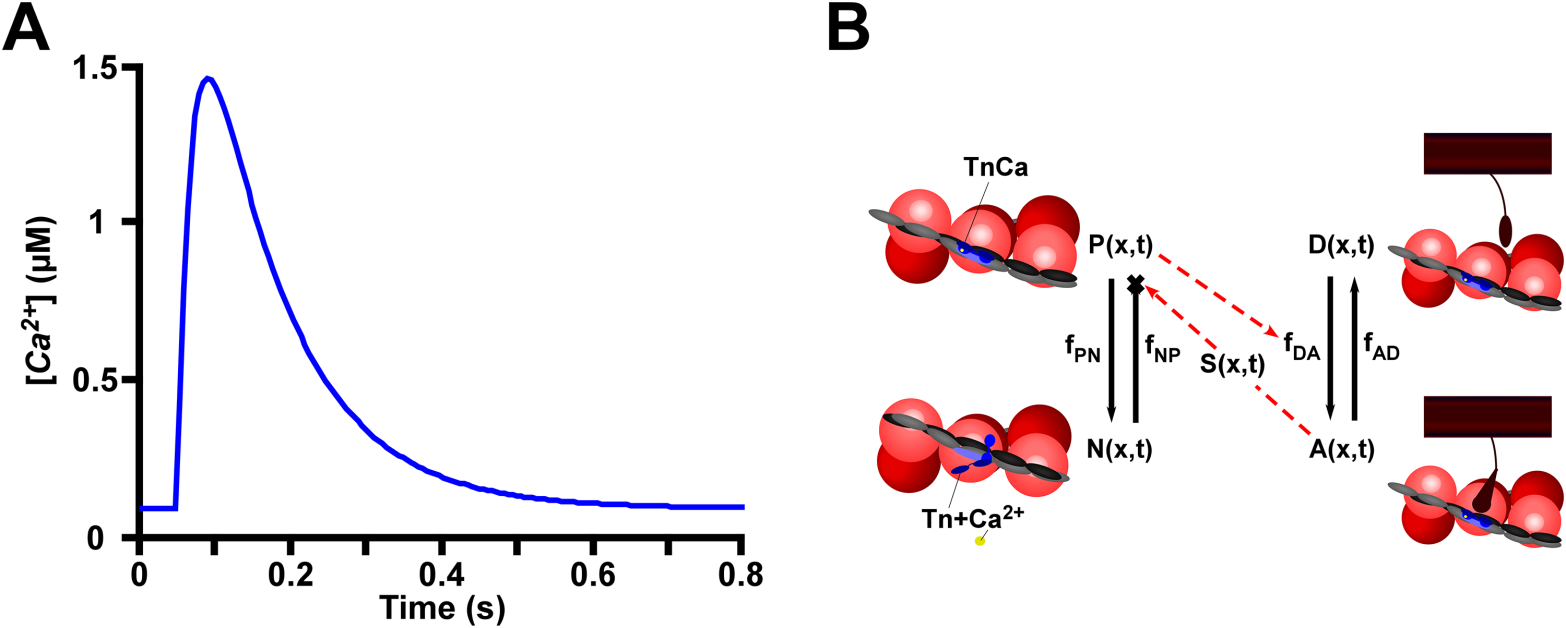
Schematic of the MechChem model. (A) The displayed calcium transient derived from Rice et al. [10] is used as an input to the model. (B). Activation of the thin filament, moving from the non-permissive state (*N*(*x*,*t*)) to the permissive state (*P*(*x*,*t*)) occurs when calcium binds to the *Tn*. Myosin heads in the detached, non-force-generating state (*D*(*x*,*t*)) can only enter the bound, force-generating cross-bridge state (*A*(*x*,*t*)) if the *XB* binding sites are free for binding. Hence, *P*(*x*,*t*) increases the rate of cross-bridge attachment (*f*_*DA*_). *A*(*x*,*t*) determines the cross-bridge force density and hence the tension (*S*(*x*,*t*)) in the thin filament. The tension suppresses the rate of detachment of *Ca*^*2+*^ from *Tn* (*f*_*NP*_). Solid arrows represent state transition rates while the red dashed arrows indicate effects on state transition rates.

In this study, we have modified the MechChem model of the cardiac sarcomere to mimic isometric twitch conditions. One of the steady state model assumptions detailed by Dupuis et al. [5] has changed, and two assumptions have been added. It is no longer assumed that unblocking of a binding site automatically implies *XB* binding and force development. Instead, a two state cross-bridge cycle model has been implemented where *Ca*^*2+*^ binding to *Tn* unblocks the binding sites, enabling *XB* formation with a given reaction rate (Fig 1B). One *new* assumption is that the intracellular *Ca*^*2+*^ concentration varies in time according to the formulation presented by Rice et al. [10] (Fig 1A). The second *new* assumption is that the cross-bridge cycle is rate-limiting in comparison with *Ca*^*2+*^ kinetics [11].

At rest, the *Tn* anchors a *Tm* molecule in place blocking the *XB* binding sites on actin monomers. The binding of a *Ca*^*2+*^ ion to the *Tn* triggers a cascade of conformational changes [12]. When the *Tn* is activated, the *Tm* strand moves so that the *XB* binding sites on the thin filament are unblocked [13]. We assume that the single overlap region is the only region on the thin filament where *XB*’s can bind. Hence, the working domain of the model is the single overlap region. The position (*x*) is defined as the position along the single overlap region starting closest to the mid line and ending toward the Z-disk. The mathematical formulation of the proportion (*P*(*x*,*t*)) of active *Tn*’s at time *t* and position *x* along the single overlap region is from the previously developed MechChem model (Eq 1). Parameters *C*_*S*_ and *K*_*TnCa0*_ represent the tension sensitivity constant of *Tn* and an equilibrium [*Ca*^*2+*^] constant, respectively.

$$P(x,t)=\frac{1}{1+e^{n(-C_{s}S(x,\;t)-ln(\frac{\left[{Ca}^{2+}](t\right)}{K_{TnCa0}}))}}$$ (1)

Parameter *n* indicates the Hill coefficient of *Ca*^*2+*^ binding to *Tn* in the absence of thin filament tension. We previously found that parameter *K*_*TnCa0*_ changes with *L*_*sarc*_ [5]. We now assume *K*_*TnCa0*_ to be linearly related to *L*_*sarc*_ (Eq 2).

$$K_{TnCa0}=-a\;L_{sarc}+b$$ (2)

Parameters *a* and *b* represent the slope and intercept of the linear relation.

The *XB* cycle model utilized in our study is based on the *XB* cycle model by Landesberg and Sideman [14]. We simplified the model (Fig 1B) to a two state model that consisted of a force generating attached state and a non-force generating state being quantified by the fractions *A*(*x*,*t*) and *D*(*x*,*t*) = 1—*A*(*x*,*t*), respectively. The rate of *XB* attachment is assumed to be proportional to the fraction *P*(*x*,*t*) of activated *Tn* complexes. Thus, for the rate of *XB* increase, the change in *A*(*x*,*t*) with respect to time is represented with Eq 3.

$$\frac{\partial A(x,t)}{\partial t}=(1-A(x,t))P(x,t)f_{DA}-A(x,t)f_{AD}$$ (3)

In the original MechChem model developed by Dupuis et al. [5], the tension in the thin filament was defined as the sum of all of the *XB* forces from the start of the single overlap region near the mid-line till the end of the single overlap region closest to the z-disk. The model represents a population of sarcomeres. Instead of explicit modeling of individual *XB*’s, *XB* force density is represented as a continuous function of position *x*, proportional with *XB* fraction *A*(*x*,*t*).

Thus, the spatial derivative of thin filament tension (∂*S*(*x*,*t*)/ ∂*x*) is represented by Eq 4.

$$\frac{\partial S(x,\;t)}{\partial x}=C_{f}A_{F}(x,t)$$ (4)

with boundary condition: *S*(0,*t*) = 0.

The constant (*C*_*f*_) represents the tension per unit length in the thin filament if all *XB*’s are active. The length of the single overlap region depends on *L*_*sarc*_ using the formulation of Rice et al. [10] that can be found in the S1 Appendix.

### Numerical implementation

Eq 3 was solved with the following method. *A*(*x*,*t*) was represented by an array of samples along the *x* axis, being used as state variables (*A*_*ix*_) that varied in time. From the state variables *A*_*ix*_, their time derivatives are calculated as follows. Tension *S*(*x*,*t*) is calculated as *S*_*ix*_ for each sample *A*_*ix*_, applying Eq 4. Thus, the numerical integration along the *x*-direction is carried out by the cumulative summing of *A*_*ix*_. From the tension samples *S*_*ix*_, *Tn*-activation *P*_*ix*_ is calculated using Eq 1. From *Tn*-activation *P*_*ix*_, the time derivative of *XB* density ∂*A*_*ix*_/∂*t* is calculated using Eq 3. Having written ∂*A*_*ix*_/∂*t* as a function of *A*_*ix*_, the related differential equation is solved by utilizing the Matlab ode23s solver.

### Parameter estimation and simulation protocol

Fig 2 from Janssen and Hunter, 1995 [8] was discretized, and data points were extracted from each curve at time points spaced every 0.02 seconds. The parameters *C*_*s*_, *C*_*f*_, *a*, and *b* have been estimated by minimizing the error between the model-generated thin filament tension (*S*_*model*_) and the experimental data from Janssen and Hunter (*S*_*experiment*_). The objective function used to calculate the error is shown in Eq 5.

$$error=\frac{\sum_{i=1}^{j}{\left(S_{model}-S_{experiment}\right)}^{2}}{j}$$ (5)

**Fig 2:**
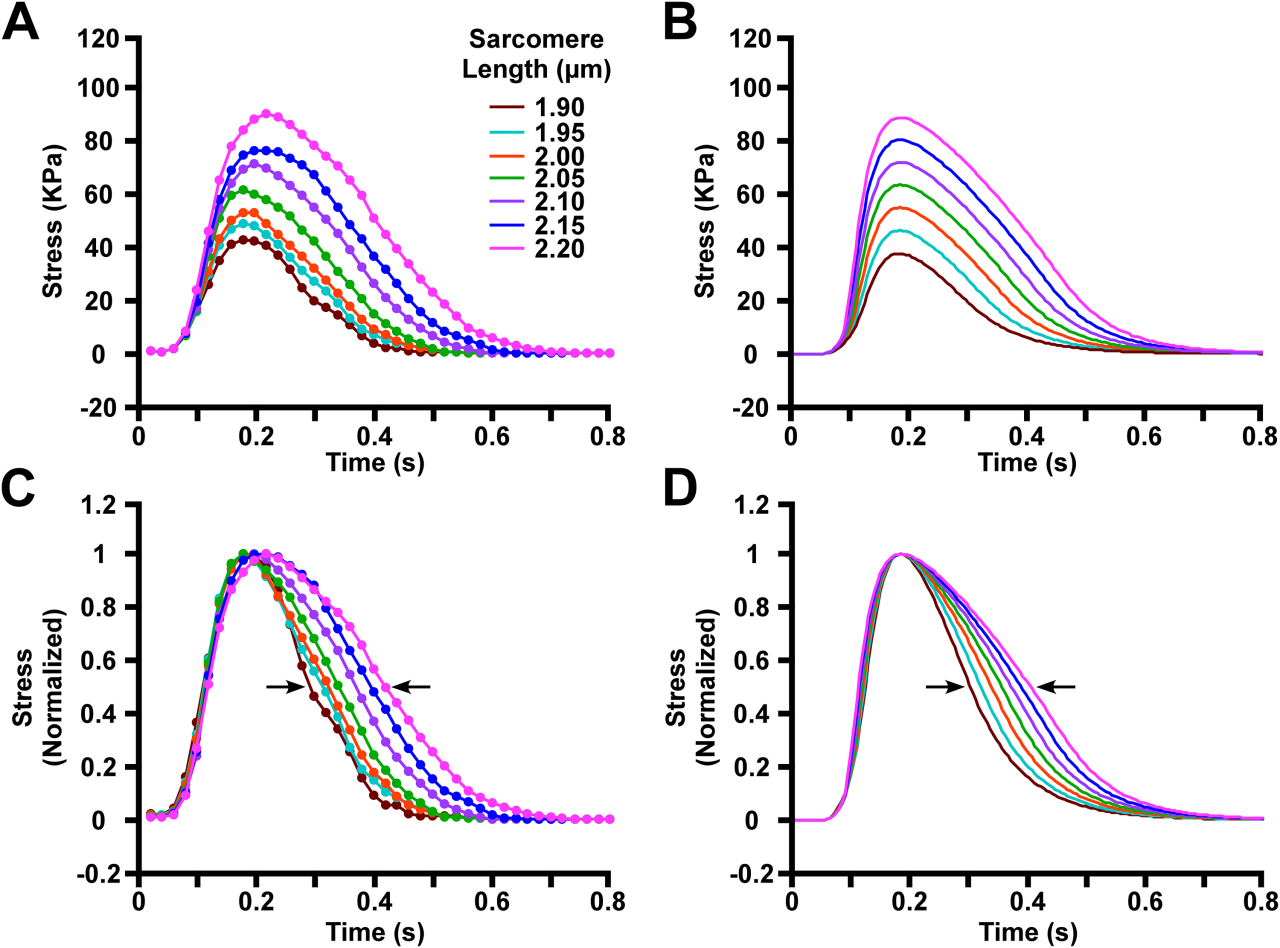
Tension traces of sarcomeres in isometric conditions. (A) Experimentally measured tension traces at *L*_*sarc*_ ranging from 1.90 to 2.20 μm under isometric conditions. The dots are data points extracted from Figure 2 of Janssen and Hunter 1995 [8]. (B) Model simulated tension traces mimicking the conditions in A are displayed. (C) The experimentally measured tension data in A are each normalized to their own peak tension. The arrows in the figure represent the relaxation to 50% of the maximum stress level after the peak stress. (D) The model-generated tension traces in B are each normalized to their own peak tension.

### Simulation protocol

Simulations were designed to mimic the isometric twitch experiments of Janssen and Hunter. *L*_*sarc*_ was held constant given parameter values *a*, *b*, *C*_*f*_, and *C*_*s*_, while calcium concentration changed in time with a peak value of 1.45 μM. The model provided thin filament tension as a function of time for 7 values of *L*_*sarc*_ ranging from 1.90 to 2.20 μm. Additionally, simulations were performed in which *L*_*sarc*_ was maintained at 2.2 μm while calcium gradients were varied as follows: the amplitude of the calcium concentration was increased stepwise from 60% to 100% of the peak value used in the other simulations while the time constants of rise and decay remained constant. This simulation protocol mimics the experiments of Kassiri et al [9], showing that twitch duration increases with peak force independently from sarcomere length.

## Results

The full set of parameters utilized is displayed in Table 1. The parameter values resulting from the fitting procedure are *a*, *b*, *C*_*f*_, and *C*_*s*_.

**Table 1:** MechChem model parameters.

Parameter	Value	Units	Equation	Source
*a*	1.39	μM μm^-1^	2	model fit
*b*	7.76	μM	2	model fit
*f*_*DA*_	40	s^-1^	3	[14]
*C*_*f*_	0.283	kPa nm^-1^	4	model fit
*C*_*S*_	0.127	kPa^-1^	1	model fit
*n*	3	unitless	1	[4]
*f*_*AD*_	12	s^-1^	3	[14]

The comparison between data points from the experimental isometric twitch tension curves of Janssen and Hunter [8] and the model-generated tension curves is shown in Fig 2A and 2B. The ratio of the peak tension at *L*_*sarc*_ 1.9 μm to that measured at *L*_*sarc*_ 2.2 μm was 0.48 in experiments and 0.42 in simulations.

**Fig 3:**
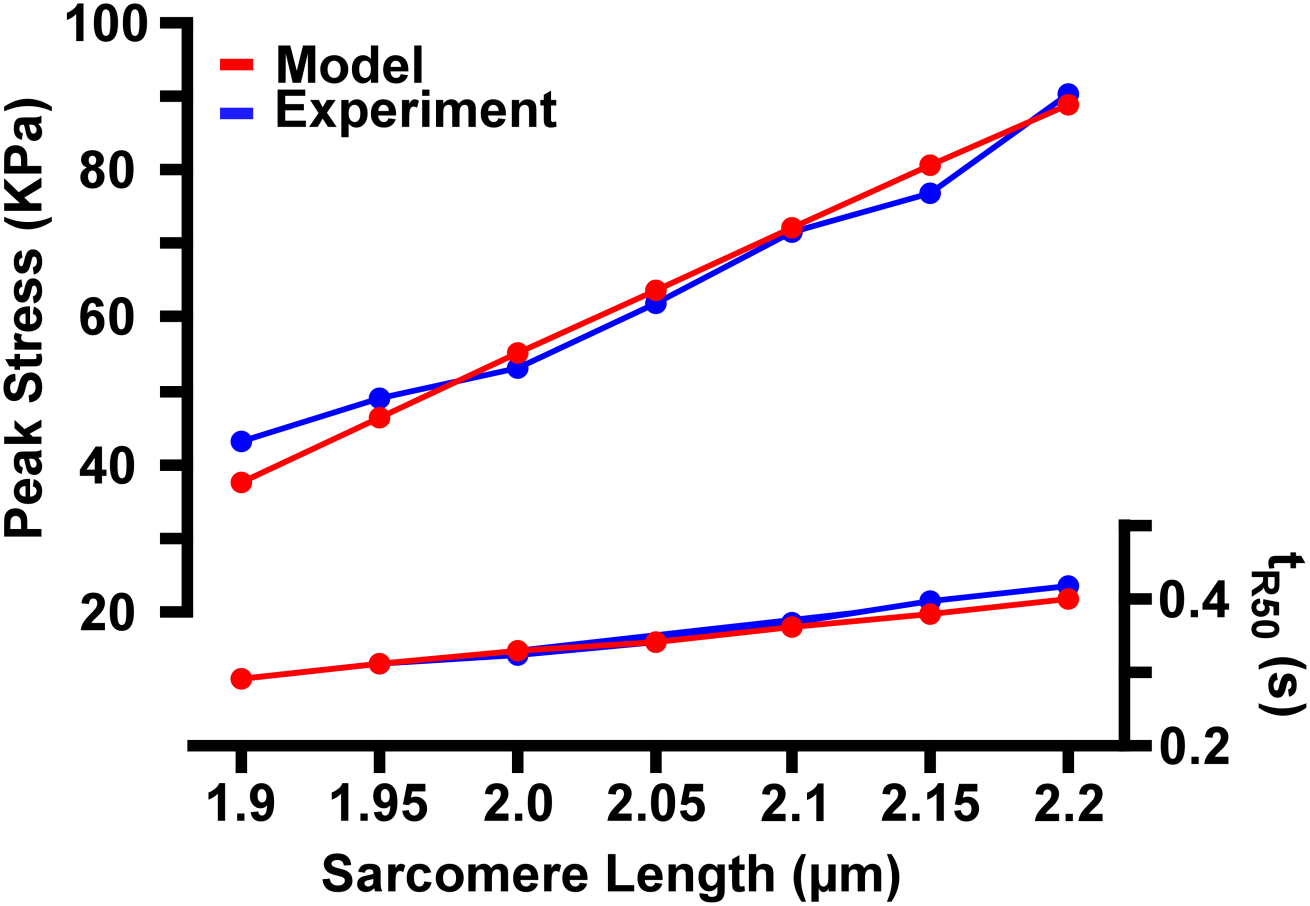
Comparison of model predicted metrics with experimental results. The peak tension and the time at which the muscle has relaxed by 50% (*t*_*R50*_) for each *L*_*sarc*_ are compared between the model prediction (red) and the experimental results (blue).

Fig 2C and 2D display the tension traces of Fig 2A and 2B, respectively, but each curve is normalized to its own peak tension. From these normalized curves, the prolongation of tension generation is obvious at larger *L*_*sarc*_’s. The time from peak tension generation to 50% relaxation (*t*_*R50*_) differs between the largest (*L*_*sarc*_ = 2.2 μm) and the smallest (*L*_*sarc*_ = 1.9 μm) *L*_*sarc*_ by 0.12 seconds in experiments and 0.11 seconds in simulations. The *t*_*R50*_ for the sarcomere lengths ranging from 1.9 μm to 2.2 μm is shown in Fig 3. Additionally, the peak tension generated by the model has been compared to the experimental values from Janssen and Hunter [8] in Fig 3.

**Fig 4:**
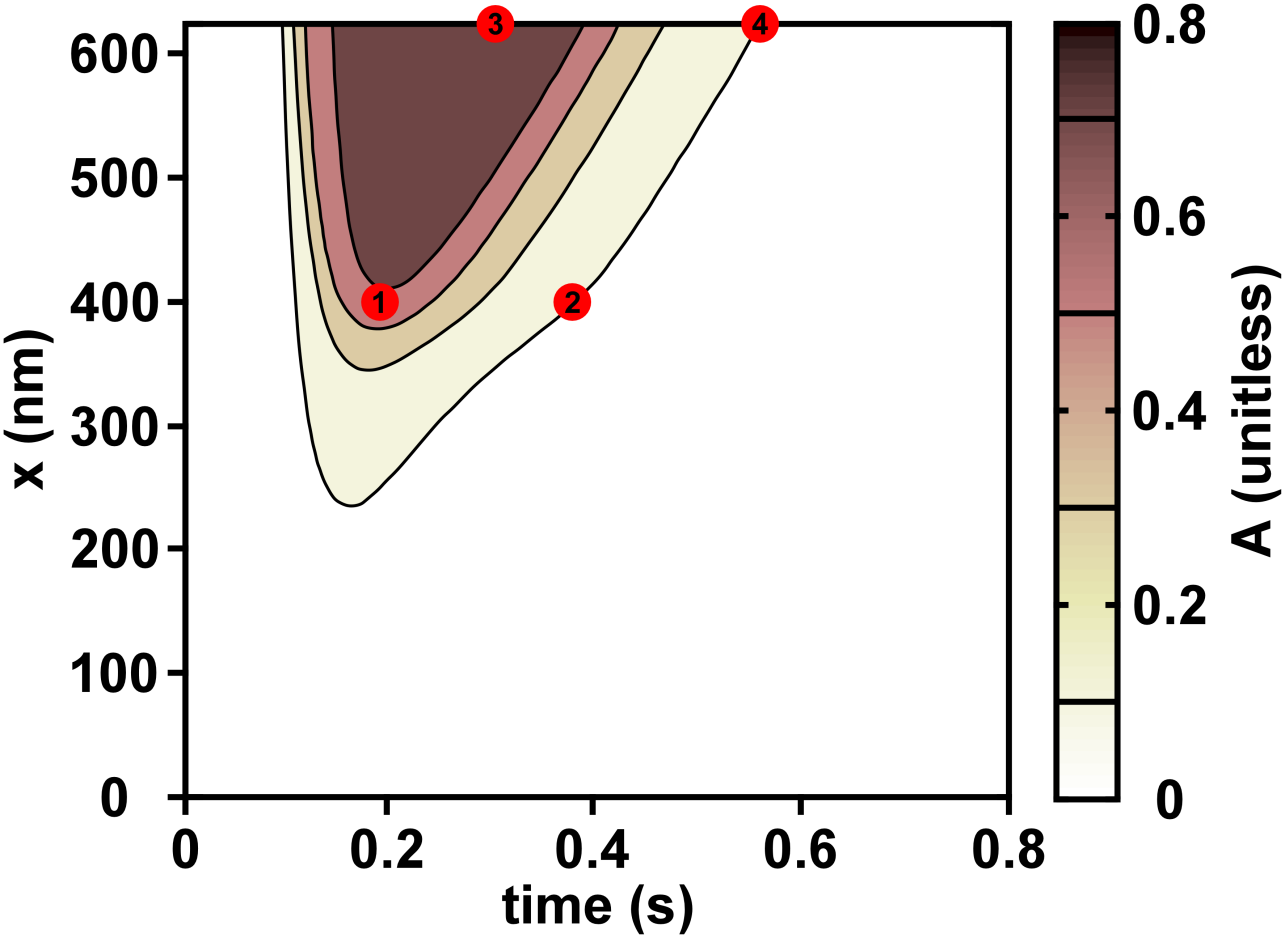
Proportion of force-generating cross-bridges (*A*) throughout an isometric twitch. *A* is displayed for sarcomere with length 2.05 μm under isometric twitch conditions. The probability of formation of a force-generating cross-bridge depends both on time and position (*x*) along the single overlap region of the thin filament. Markers 1 and 2, located at 400 nm from the mid line, show the peak level of activation of 0.65 and when the activation level reaches 0.10, respectively. Markers 3 and 4, located at 625 nm from the mid line closest to the z-disk, show the peak level of activation 0.77 and the subsequent drop to 0.10, respectively.

The model predicted proportion of *XB*’s in the force generating state (*A*) during an isometric twitch as a function of time (*t*) and position (*x*) at *L*_*sarc*_ = 2.05 μm is shown in Fig 4. The *L*_*sarc*_ of 2.05 μm was chosen because it was in the middle of the range of *L*_*sarc*_’s. The model predicts that the highest likelihood of *XB*’s to generate force occurs for high *x*-values, i.e. in the single overlap region closest to the z-disk. Tension development at position *x* in the thin filament is prolonged in the single overlap region near the z-disk as compared to this region near the center of the sarcomere. At *x* = 400 nm, the probability that there will be a strong cross-bridge bound reaches its peak value at a time of 0.20 s (marker 1). The probability of a bound cross-bridge drops to 0.10 by 0.38 s (marker 2). However, at the boundary of the single overlap region near the z-disk (*x* = 625 nm), the probability of cross-bridge formation reaches its peak value of 0.77 at 0.31 s (marker 3) and drops to 0.10 at 0.56 s (marker 4). The time difference between markers 2 and 4 shows that relaxation of the thin filament is delayed in the area under the highest tension, i.e. in the single overlap region near the z-disk.

**Fig 5:**
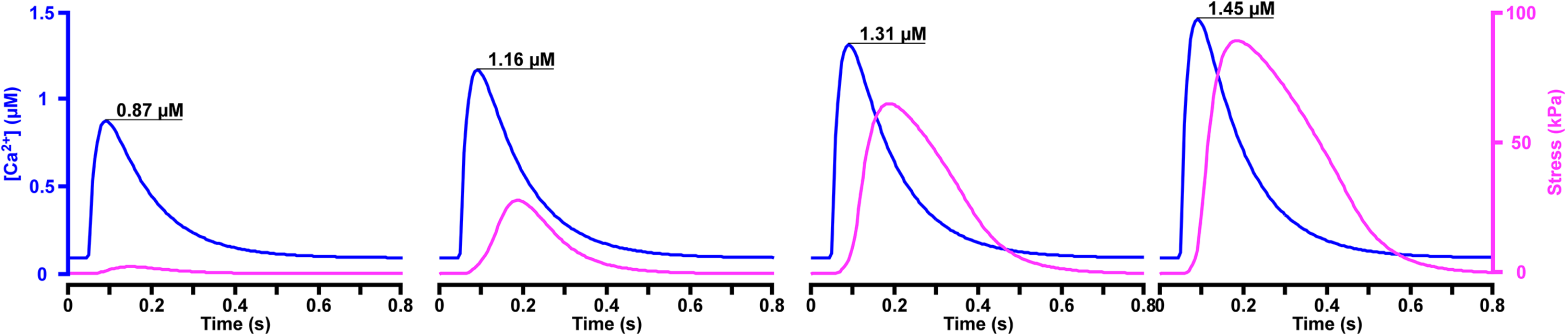
Isometric twitch stress with increases in peak [*Ca*^*2+*^]. The input calcium transient (blue curves) increases its peak value from 0.87 μM to 1.45 μM while maintaining time constants of rise and decay. The related isometric twitch stress curves (magenta) are shown for a sarcomere length of 2.2 μm.

Fig 5 displays myofiber isometric twitch stress curves (magenta) resulting from input calcium transients (blue). The peak intracellular calcium concentration was increased from 0.87 μM to 1.45 μM. The *t*_*R50*_ for the lowest peak intracellular calcium concentration was 0.10 s, increasing to 0.22 s when the peak intracellular calcium concentration was increased to 1.45 μM. With greater peak tension, the duration of contraction increased.

## Discussion

To study the time course of force development and relaxation of cardiac sarcomere contraction, we incorporated the dynamics of the cross-bridge cycle in our previously developed MechChem model of the mechano-chemical interactions in cardiac troponin activation [5]. In addition to the assumed tension-dependent calcium-binding cooperativity in this model, we implemented time dependence by means of imposed calcium transients. We simulated isometric twitches for a range of sarcomere lengths and compared our results to the experimental data of Janssen and Hunter and Kassiri et al. [8, 9]. Our model with only six parameters and two partial differential equations could reproduce the isometric twitch behavior as evidenced by strikingly similar values for each sarcomere length between model and experiment for peak tension, time of 50% relaxation, and increases in twitch duration with increased peak intracellular calcium concentration. These results not only suggest that the resultant force development after binding of calcium to troponin favors new bindings of calcium to troponin, but also that hindrance to thin filament deactivation increases with tension in the thin filament. The resultant of both mechanisms is an increase of both peak isometric tension and duration of the twitch with sarcomere length and peak intracellular calcium concentration. An interesting consequence of our hypothesis on tension-dependency of calcium binding to troponin is that contraction duration is locally distributed within a sarcomere, with longest duration close to the z-disk where tension in the thin filament is highest.

### Intracellular calcium transient

There are multiple channels, pumps, and buffers that determine the intracellular calcium transient in cardiac muscle [1]. For simplicity we have utilized the prescribed calcium transient formulated by Rice et al. [10] as the input in this study. While calcium handling is tightly controlled in cardiac muscle, there are pathological situations that cause differences in calcium transient morphology [15]. A model of the calcium handling system will be necessary when studying a pathological situation, but the prescribed transient is suitable for our study in which we investigate the isometric twitch in healthy tissue at different sarcomere lengths. While a change in sarcomere length has an acute effect on the calcium transient, a recent article [16] has shown that intracellular calcium transient morphology does not exhibit significant long term changes in rat ventricular myocytes with a change in cell length.

### Rise of tension

Experiments have shown that the upslope of tension in isometric twitches decreases with sarcomere length [8, 17]. The MechChem model simulated upslope of tension development is independent of sarcomere length. It is possible that addition of a third cross-bridge state, a weakly-bound, non-force-generating state, could alter the rate of rise of tension in the sarcomere. It is also possible that the assumed calcium transient morphology is inconsistent with that in the experiments of Janssen and Hunter [8]. Unfortunately, the free intracellular calcium concentration was not measured in the experiments. Janssen and Hunter altered the extracellular calcium concentration and did not measure the intracellular concentrations of calcium. Hence, we assumed a calcium transient in the absence of exact measurements.

### Linear increase of peak tension with sarcomere length

Experiments have shown that the peak isometric twitch tension developed in the sarcomere increases with sarcomere length [8, 18]. The results of the MechChem model show a similar linear relationship between peak tension and sarcomere length. The increase in peak tension with sarcomere length shown in our simulations is a result of the increasing length of the single overlap region and the mechanism of cooperative activation as described by the MechChem model [5]. We are aware that there are many proposed mechanisms of cooperative activation in cardiac muscle including nearest neighbor interactions [19], myofilament lattice spacing [20], the cooperative realignment of binding sites [21], the interaction between mutually overlapping *Tm*’s under *Tn*’s [22], and the positive feedback between the number of cross-bridges and the binding affinity of *Ca*^*2+*^ for *Tn* [23], just to name a few. We previously proposed that the affinity of *Ca*^*2+*^ binding to *Tn* is boosted by mechanical tension locally in the thin filament. The cooperative mechanism we proposed is consistent with the results of previous studies suggesting that *Ca*^*2+*^ binding to *Tn* tightens with sarcomere tension [17]. Hence, the cooperative activation mechanism presented in our previous study [5] was conserved within the current model framework. However, parameters *C*_*s*_ and *C*_*f*_ changed values because of the different experimental circumstances [3]. With the addition of the *XB* cycle, we have presented a model of the cardiac sarcomere that is composed of 2 partial differential equations and six parameters that is able to reproduce a range of isometric twitch conditions without further change of parameter values.

### Increase of peak tension with peak intracellular calcium concentration

Increases in peak intracellular calcium concentration result in increases in peak myofiber tension in both steady state isometric experiments [2] and isometric twitch experiments [9]. In the MechChem model, the affinity of *Tn* to bind *Ca*^*2+*^ increases with *L*_*sarc*_. Therefore, some of the increases in twitch duration shown at high *L*_*sarc*_ can be due to the increased affinity for *Ca*^*2+*^ instead of solely due to the increased peak tension. To uncouple the increase in tension generation from increased *Ca*^*2+*^ affinity, we have mimicked the experimental conditions of Kassiri et al. by maintaining the *L*_*sarc*_ at 2.2 μm while altering the peak intracellular calcium concentration. The working range of calcium concentrations used by Kassiri et al. in experiments was lower than that used in our model, so relative changes were utilized for comparison, and the amplitude of the intracellular calcium transient was increased stepwise from 60% to 100% of the peak value used in our simulations. With greater peak intracellular calcium concentration, tension in the thin filament increased. The twitch duration increased with peak tension independent of the *L*_*sarc*_ in these simulations. The additional test of the model provides further results supporting our hypothesis that greater tension in the thin filament locally hinders relaxation.

### Tension dependent mechanism of relaxation in cardiac muscle

The experiments of Janssen and Hunter [8] have shown that cardiac myocyte relaxation during an isometric twitch is prolonged with greater developed peak tension. However, a likely physical mechanism that causes the prolongation of contraction is not described yet. A recent review by Biesiadecki et al. [24] highlights three possible rates that determine the moment and rate of relaxation: intracellular calcium decline, troponin deactivation, and cross-bridge cycling rates. Each of the three rates mentioned contribute to relaxation morphology.

Many computational models have been developed that replicate the prolongation of contraction with greater peak tension generated in the cardiac sarcomere. Landesberg and Sideman [14] developed a model of the cardiac sarcomere including *Tn* activation and the *XB* cycle. They propose that the number of strongly bound *XB*’s increases the affinity of *Tn* to bind *Ca*^*2+*^. Similarly, Niederer et al. proposed a thorough mathematical description of the cardiac sarcomere to understand relaxation of cardiac muscle [25]. The tension developed in the sarcomere by the Niederer model decreases the rate of detachment of *Ca*^*2+*^ from *Tn*. While both the Niederer and the Landesberg and Sideman models propose cooperativity mechanisms dependent on sarcomere tension, an underlying physical explanation is lacking. They claim interactions between *Tn*’s on the thin filaments over distances as large as 35 nm but do not provide a firm physical basis for this interaction. With the MechChem model, we propose a physico-chemical mechanism that tightens *Ca*^*2+*^ binding to *Tn* by molecular deformation caused by mechanical tension in the thin filaments, finally resulting in prolongation of contraction at higher peak tension. By incorporating the spatial scale along the thin filament, tension in the thin filament changes locally with position *x*. Greater tension in the thin filament renders release of *Ca*^*2+*^ from *Tn* energetically less favorable. Consequently, high tension in the thin filament impedes relaxation.

The model developed by Rice et al. [10] is able to reproduce sarcomere mechanics including isometric twitches and isotonic contractions utilizing a system of ordinary differential equations (ODE). The prolongation of contraction at higher *L*_*sarc*_’s is present within the results of the Rice model. The latter authors assume a mathematical formulation that the parameter governing the rate of transition from the weakly bound, non-force-generating *XB* state to the unbound *XB* state increases at lower *L*_*sarc*_’s. Consequently, the prolongation of contraction is, at least in part, prescribed by the parameter formulation. However, the mechanism behind the increasing rate transition rate at lower *L*_*sarc*_’s is unclear.

We propose that high tension increases the strength of the binding between *Ca*^*2+*^ and *Tn*, thereby hindering relaxation. In our simulations, relaxation begins in areas of the single overlap region closer to the midline where tension is lower. Even if tension in the thin filament at the z-disk is at its maximum, there is always a relatively loose end closest to the sarcomere mid line under no tension. Consequently, as the intracellular calcium concentration decreases, relaxation will begin at this loose end first.

Our model simulations suggest heterogeneity in *XB* binding along the single overlap region caused by the gradient of tension along the thin filament. This directionality in *XB* binding was not found by Desai and colleagues [26], who fluorescently labeled S1 myosin heads to view individual bindings to an actin monomer on a strand. However, the myosin heads in that study were free in solution, so there was no force generated. According to our hypothesis that thin filament tension enhances *Ca*^*2+*^ binding to *Tn*, we would not expect directionality to cross-bridge binding when the thin filament is not subject to mechanical load. The hypothesis we propose could potentially be tested if the experimental setup of Desai could be altered to incorporate force generating myosin heads.

### Limitations

The MechChem model is a relatively simple model that utilizes two partial differential equations and six parameters to characterize the isometric twitch contraction of a cardiac sarcomere. Due to the simplicity of the MechChem model, some mechanisms that impact the cooperative binding of *Ca*^*2+*^ to *Tn* may not be included. Additionally, a prescribed calcium transient has been imposed as an input to the model, so force feedback on the electrophysiology [27] is currently not included. However, the calcium transient is an input that can later be replaced with a model. In the study by Janssen and Hunter [8] intracellular [*Ca*^*2+*^] was not measured. Hence, we had to make assumptions on the calcium transient. Additionally, we have utilized the assumption that [*Ca*^*2+*^] is uniform throughout the entire sarcomere lattice and the cell. It has been shown that in large animals such as pigs, there are intracellular spatial inhomogeneities in the *Ca*^*2+*^ transient [28]. However, mice did not display inhomogeneity in [*Ca*^*2+*^] probably due to the high density of T-tubules. Hence, the assumption of [*Ca*^*2+*^] homogeneity is likely relatively accurate when modeling rat cardiomyocytes but may be incorrect when modeling a larger animal or human. Additionally, the calcium dynamics controlling the activation of the thin filament in the MechChem model are currently represented with the steady state calculation of *P*(*x*,*t*). The use of steady state calcium binding rather than a dynamic description is considered a reasonable assumption because the cross-bridge cycle has been shown to be rate limiting. Therefore, the calcium dynamics can be viewed in equilibrium. When sarcomere shortening is added to the model, a description of the dynamics of calcium binding will be necessary.

The MechChem *XB* cycle model is a simplified, two state model. Although the two-state *XB* cycle used in our model is a simplified representation of a more complex biological system, the lumping of these states improves the computational expense of running such a model while still providing sufficient complexity necessary to test our hypothesis.

### Conclusion

The MechChem model of cardiac sarcomere contraction has been extended with a *XB* cycle model and a calcium transient that changes in time to simulate an isometric twitch at multiple sarcomere lengths. The results of the MechChem model showed that peak isometric twitch tension and the duration of the twitch increased with sarcomere length. The results support our hypothesis that high tension in the thin filament locally hinders relaxation. Compared to other models of cardiac sarcomere isometric twitch contraction, the MechChem model is simple with few parameters while many properties of myocyte contraction are included.

## Supporting information

S1 Appendix Calculation of the calcium transient and single overlap region length. The equations used to calculate the prescribed calcium transient and the length of the single overlap region are described.
(DOCX)

S1 Model Code Zip file containing the Matlab program representing the MechChem model. (ZIP)
