# Supplementary material for: High tension in sarcomeres hinders myocardial relaxation: A computational study
Source: PLoS One. 2018 Oct 4;13(10):e0204642. doi: 10.1371/journal.pone.0204642 (PMC6171862; doi:10.1371/journal.pone.0204642)
Supplement: S1 Appendix — The equations used to calculate the prescribed calcium transient and the length of the single overlap region are described. (DOCX) [file pone.0204642.s001.docx]

## S1 Appendix: Calculation of the calcium transient and single overlap region length

## Calcium Transient

The calcium transient imposed in model simulations is determined by the formulation of Rice et al. (1). Equations A1 and A2 are utilized to represent the transient [*Ca^2+^*] with time constants *τ_1_* and *τ_2_*, resting levels of [*Ca^2+^*] (*C_dias_*), and the maximum amplitude of the calcium transient (*C_amp_*).

| $\beta= \left( \frac{\tau_{1}}{\tau_{2}} \right)^{\frac{-1}{\frac{\tau_{1}}{\tau_{2}}-1}}-\left( \frac{\tau_{1}}{\tau_{2}} \right)^{\frac{-1}{1-\frac{\tau_{2}}{\tau_{1}}}}$ | (A1) |
| --- | --- |

| $\left[ {Ca}^{2+} \right]\left( t \right)=\left( \frac{C_{amp}-C_{dias}}{\beta} \right)\left( e^{-\frac{t}{\tau_{1}}}-e^{-\frac{t}{\tau_{2}}} \right)+C_{dias}$ | (A2) |
| --- | --- |

## The parameter values utilized to determine the calcium concentration are as follows: C_amp_ = 1.45 µM, C_dias_ = 0.09 µM, τ_1_ = 0.02 s, and τ_2_ = 0.11 s.

## Single Overlap length

The length of the single overlap region (*x_max_*) between thick and thin filaments is determined by sarcomere length (*L_sarc_*), thin filament length (*L_thin_*), thick filament length (*L_thick_*), and the length of the bare zone (*L_bare_*). The formulation of Rice et al (1) is used to calculate the length of the single overlap region:

| $x_{max}=x_{z}-x_{c}$ | (A3) |
| --- | --- |

| $x_{z}=min\left( \frac{L_{thick}}{2}, \frac{L_{sarc}}{2} \right)$ | (A4) |
| --- | --- |

| $x_{c}=max\left( \frac{L_{sarc}}{2}- \left( L_{sarc}-L_{thin} \right), \frac{L_{bare}}{2} \right)$ | (A5) |
| --- | --- |

The values utilized to compute the length of the single overlap region are as follows: *L_thin_* = 1.2 µm, *L_bare_* = 0.1 µm, *L_thick_* = 1.6 µm (2).

**References**

1. Rice JJ, Wang F, Bers DM, de Tombe PP. Approximate Model of Cooperative Activation and Crossbridge Cycling in Cardiac Muscle Using Ordinary Differential Equations. Biophysical Journal. 2008;95(5):2368-90.

2. Poole DC, Lieber RL, Mathieu-Costello O. Myosin and actin filament lengths in diaphragms from emphysematous hamsters. Journal of applied physiology (Bethesda, Md : 1985). 1994;76(3):1220-5.
